# Resistance training for metabolic dysfunction-associated steatotic liver disease:a systematic review and meta-analysis

**DOI:** 10.3389/fphys.2025.1679094

**Published:** 2026-02-02

**Authors:** Yun Chen, Xiaoya Qiao, Xinyi Zou, Kai Xu, Zhongke Gu, Gangrui Chen, Jiansong Dai

**Affiliations:** 1 Department of Sport and Health Sciences, Nanjing Sport Institute, Nanjing, China; 2 Sport Science Research Institute, Nanjing Sport Institute, Nanjing, China

**Keywords:** resistance training, MASLD, randomized controlled trial, exercise, metabolic dysfunction associated steatotic liver disease

## Abstract

**Objective:**

The aim is to systematically assess the impact of resistance exercise on patients diagnosed with metabolic dysfunction-associated steatotic liver disease (MASLD).

**Methods:**

Randomized controlled trials (RCTs) focusing on resistance exercise for MASLD/NAFLD were sourced from PubMed, Embase, the Cochrane Library, Web of Science, Scopus, and CNKI databases, (covering all entries from inception to 18 March 2025. The Cochrane Risk of Bias Tool was utilized for quality evaluation, and RevMan 5.3 software was used for conducting the meta-analysis.

**Results:**

Eleven RCTs, involving 395 participants, were included in the analysis. The meta-analysis revealed that alanine aminotransferase (ALT) levels were significantly decreased (MD = −4.44 U/L, 95% CI = −8.84 to −0.03, Z = 1.98, P = 0.05). No significant difference was observed in aspartate aminotransferase (AST) levels (MD = −0.18 U/L, 95% CI = −6.70 to 6.34, Z = 0.05, P = 0.96). Among the eight imaging assessment studies, seven reported substantial reductions in liver fat content, whereas one study indicated no effect. No significant difference in clinical compliance was detected between the groups (RR = 0.99, 95% CI = 0.89 to 1.10, P = 0.92), and no serious adverse events were documented.

**Conclusion:**

Resistance exercise notably enhances ALT levels and hepatic steatosis in patients with MASLD. The recommended minimum effective dosage is whole-body multi-muscle training, consisting of 8–10 exercises at 60%–80% of one-repetition maximum (1RM) intensity, performed at least three times weekly for a minimum of 12 weeks. This intervention is particularly advantageous for patients with elevated ALT levels who are unable to tolerate aerobic exercise, and it appears to be safe. Nonetheless, further research involving larger sample sizes is required to confirm its long-term efficacy.

**Systematic Review Registration:**

https://www.crd.york.ac.uk/PROSPERO/view/CRD420251050504, identifier CRD420251050504.

## Introduction

Metabolic dysfunction-associated steatotic liver disease (MASLD) has become the most common chronic liver disease globally, affecting about 20% of the global adult population (Younossi et al., 2025). In China, 29.2% of the population suffers with MASLD, with a greater prevalence rate among the elderly population (Liang et al., 2022). In addition to being closely linked to higher risks of cirrhosis and liver damage, metabolic dysfunction-associated steatotic liver disease (MASLD) may also worsen metabolic syndrome (Van Nieuwenhove et al., 2021), raise the risk of chronic kidney disease (Bilson et al., 2024), cardiovascular disease (Wong et al., 2011), and cancer (Huang et al., 2021).

Therefore, creating evidence-based exercise therapies for MASLD is crucial. Currently, pharmacological research for MASLD is still in the clinical development phase, and the major therapy approach remains lifestyle modification (Chai et al., 2023), which generally consists of weight loss, dietary changes, and moderate exercise (Zhang et al., 2025). Following a Mediterranean diet or losing 5% of one’s body weight can help reduce liver fat (Romero-Gómez et al., 2017). Appropriate exercise can improve MASLD patients’ quality of life, cardiorespiratory function, visceral fat loss, and muscle quality and function (Zeng et al., 2024). However, different types of exercise have variable impacts on MASLD.

In order to improve muscle strength, mass, and bone density, resistance training entails having muscles resist external resistance. Additionally, resistance exercise improves insulin resistance, hypertension, dyslipidemia, and bone density (Hallsworth et al., 2011; Lemes et al., 2016; Castaneda et al., 2002). Resistance training is also an option for MASLD patients who are unable to engage in aerobic exercise or who have impaired cardiorespiratory function (Whitsett and VanWagner, 2015). Resistance training does have certain drawbacks, though, like the requirement for specialized facilities, equipment, and exercise techniques. Resistance training has particular benefits on metabolic-related conditions, even though current clinical guidelines require 150 min of moderate-intensity aerobic exercise per week to significantly improve liver fat (Tacke et al., 2024).

Resistance training has been shown to improve insulin sensitivity, muscle mass, and metabolic health, which are particularly relevant to MASLD pathophysiology. However, unlike aerobic exercise, the effects of resistance training on liver-specific outcomes—such as hepatic steatosis, inflammation, and fibrosis—remain less established. Current clinical guidelines emphasize aerobic exercise, yet a significant proportion of MASLD patients may be unable to engage in sustained aerobic activities due to comorbidities such as obesity, osteoarthritis, or cardiorespiratory limitations. Thus, resistance training represents a viable alternative exercise modality for this population. Despite its potential, several unknowns persist regarding resistance training in MASLD:Its efficacy in reducing liver fibrosis has not been adequately studied. The optimal dose, frequency, and duration of resistance training for MASLD are not well-defined. Its long-term effects on liver histology and metabolic parameters remain unclear. Whether resistance training can independently improve outcomes beyond weight loss or dietary changes is still uncertain.

The main purpose of this review is to explore the effects of resistance training on liver fat in MASLD patients. Given the changing terminology, and the fact that the majority of the included studies were published prior to the transition, we used “NAFLD”, “MAFLD” and “MASLD” throughout the review to provide consistency. We carried out a thorough systematic review to determine the effects of resistance exercise interventions on liver fat in MASLD patients, elucidating the role of resistance exercise in metabolic dysfunction-associated steatotic liver disease and providing a theoretical foundation for developing resistance exercise interventions for MASLD patients.

## Methods

We performed a comprehensive review using the Cochrane Guidelines for comprehensive Reviews of Interventions (Higgins et al., 2024). In our synthesis, we followed the PRISMA criteria (Liberati et al., 2009). The protocol is included in the accompanying document labeled S1 file. We examined studies that studied the effects of resistance exercise therapies on liver outcome metrics in MASLD patients.

## Study type

We considered English-language full-text publications of interventional research, such as randomized controlled trials (RCTs) and non-controlled trials published in peer-reviewed journals. Studies were only considered for inclusion if they reported results relevant to the review aims.

## Participant type

In accordance with clinical recommendations for screening for the risk of fatty liver disease, we included studies assessing patients who had been diagnosed with MASLD (Tacke et al., 2024). It is crucial to remember that changing the name from NAFLD to MASLD has no bearing on the requirements for inclusion because the terminology change represents a conceptual shift rather than a modification of the essential information.

## Intervention type

Interventions that involved several training sessions and were characterized as strength- or resistance-based exercise training were included. Studies that offered integrated exercise training (with both aerobic and resistance components) or that assessed participants following a single training session (i.e., no training program) were not included. A group that did not exercise, got a phony exercise intervention, or engaged in other types of exercise was referred to as the control group. Co-interventions such motivational interviews, psychological counseling, or nutritional counseling were allowed as long as they were applied to the control group to the same degree.

## Types of outcome measures

This study looked at three key clinical outcome measures: liver biochemical markers of hepatic steatosis, quantitative imaging assessments, and liver histology investigations. These markers were chosen because to their high clinical significance, universal acceptance in clinical trials, and recommendation status in current MASLD diagnostic and therapy guidelines.

Liver biochemical indicators, particularly serum alanine aminotransferase (ALT) and gamma-glutamyl transferase (GGT) values, are often used indications of liver injury and dysfunction. Previous research has shown that changes in the activity of these enzymes are significantly associated with improvements or deterioration in liver histology (including steatosis and inflammation severity) in NAFLD patients (Kramer et al., 2021), making them important non-invasive indicators of liver health and treatment response.

Magnetic resonance imaging (MRI) and its spectrum analysis (MRS) are preferred imaging techniques because of their ability to give precise, non-invasive quantitative assessments of liver fat levels. MRI technology has been confirmed by histological gold standards and has shown superior sensitivity and specificity in identifying and measuring hepatic steatosis (Pasanta et al., 2021; Loomba et al., 2020), making it a viable tool for monitoring liver fat changes during therapy. Traditional ultrasound examinations, on the other hand, were excluded from this study’s evaluation system due to limitations such as insufficient sensitivity, high operator dependency, and a lack of precise quantification capabilities (Kramer et al., 2021; Bonekamp et al., 2014),all of which fail to meet the criteria for efficacy evaluation.

The gold standard for identifying MASLD and thoroughly evaluating liver pathological alterations (such as steatosis, inflammatory activity, and the degree of fibrosis) is still liver histological testing (Vos et al., 2017). Histological data are invaluable when the most specific information regarding liver changes is needed, despite the fact that the technique is invasive. These evaluation techniques guarantee both high reliability and obvious clinical significance for the findings on the effectiveness of resistance exercise intervention for MASLD patients that are given in this review.

## Search methods used to identify studies

### Data synthesis and statistical analysis

We conducted meta-analyses using Review Manager (RevMan) software, version 5.3. For continuous outcome measures (e.g., ALT, AST, liver fat content), the mean difference (MD) with 95% confidence intervals (CI) was calculated using the inverse variance method. For the dichotomous outcome of clinical compliance, we calculated the risk ratio (RR) with 95% CI. Statistical heterogeneity among the included studies was assessed using the I^2^ statistic and the Chi^2^ test. An I^2^ value greater than 50% was considered to indicate substantial heterogeneity. A fixed-effect model was applied when heterogeneity was not significant (I^2^ ≤ 50%); otherwise, a random-effects model was used. We had planned to perform subgroup analyses to explore potential sources of heterogeneity (e.g., intervention duration, presence of dietary co-intervention); however, this was not feasible due to the limited number of studies available for each outcome. Sensitivity analyses were conducted by sequentially removing each study to assess the robustness of the pooled results. Publication bias was planned to be assessed visually using funnel plots if a sufficient number of studies (n ≥ 10) were included in a meta-analysis.

### Electronic search

On 18 March 2025, we searched the following electronic databases: CNKI, Web of Science, Embase, PubMed, Cochrane, and Scopus. We did not restrict the research’ date or location, however we did limit the search to English publications.

### Data collection and synthesis

#### Research selection

The search results were entered into Endnote, and duplicates were deleted. Two review writers (Chen Yun and Qiao Xiaoya) separately evaluated the titles and abstracts for potential inclusion, with any disputes resolved through discussion and arbitration by a third review author (Zou Xinyi), as needed. Following the initial screening phase, we retrieved full-text articles from potentially eligible trials and used the same criteria as for titles and abstracts to determine their eligibility for inclusion using our predefined criteria. Detailed justifications for excluding full-text articles were documented. The selection process was extensively documented and illustrated with a PRISMA flow diagram.

#### Data extraction and management

The Cochrane Data Extraction and Collection Form served as the model for our standardized data extraction form, which we employed to guarantee accurate and dependable data extraction in MASLD (Smith et al., 2024). Study, participant, and outcome data were separately gathered by the two review writers, Chen Yun and Qiao Xiaoya. Discussions were used to settle disagreements, and where needed, a third review author (Zou Xinyi) was consulted.

From every study, we extracted data in multiple categories. Study title, study ID, authors, year of publication, journal or source, and nation of origin were among the general details. The disease under investigation, the study design, the date, the length of participation, and the setting were all considered study features. Participant characteristics included the following: age range, mean age, gender, ethnicity/race, height, weight, BMI, liver fat, ALT, aspartate transaminase (AST), GGT, diagnosis, comorbidities, cluster use (if applicable), recruitment methods, sample size, randomization methods, baseline imbalances between groups, and withdrawals and exclusions. The number of participants who were randomly assigned to each group was noted, along with the type, frequency, intensity, length of each training session, length of the treatment period, co-interventions, integrity of delivery (whether supervised or prescribed), and adherence. Primary and secondary outcomes, measurement timepoints, evaluation techniques, and information on the statistical methods employed were all included in the result data. Lastly, we gathered any additional data, such as the study authors’ main conclusions. Following data extraction, we arranged and synthesized the data according to the study’s design and findings. We presented the absolute mean difference for each identified outcome and gave a thorough overview for each trial. Additionally, we produced tables that summarized study results, exercise regimens, and baseline participant characteristics. Subgroup analyses were neither planned nor carried out because of the small number of papers on this subject.

#### Assessment of risk of bias in included studies

According to the Cochrane Handbook for Systematic Reviews of Interventions, two review writers, Chen Yun and Qiao Xiaoya, independently evaluated every trial using the Cochrane Randomized Trials Risk of Bias Tool. A third reviewer, Zou Xinyi, evaluated the paper independently if there were any disagreements. To guarantee uniformity across all evaluations, the writing group as a whole deliberated on final decisions. Every decision was taken in accordance with the Cochrane Handbook for Systematic Reviews of Interventions’ recommendations (Higgins et al., 2024).

#### Comparative studies providing clinical characteristics of MASLD patients

To determine the generalizability of the research included in our evaluation to the clinical MASLD population, we looked at clinical trials with biopsy-confirmed MASLD. We produced tables to summarize and compare the baseline characteristics of biopsy-confirmed MASLD to those stated in the articles included in our evaluation. We looked at individuals’ gender, mean age, ALT, AST, and GGT levels, as well as liver fat measurements.

## Results

### Search results

**FIGURE 1:**
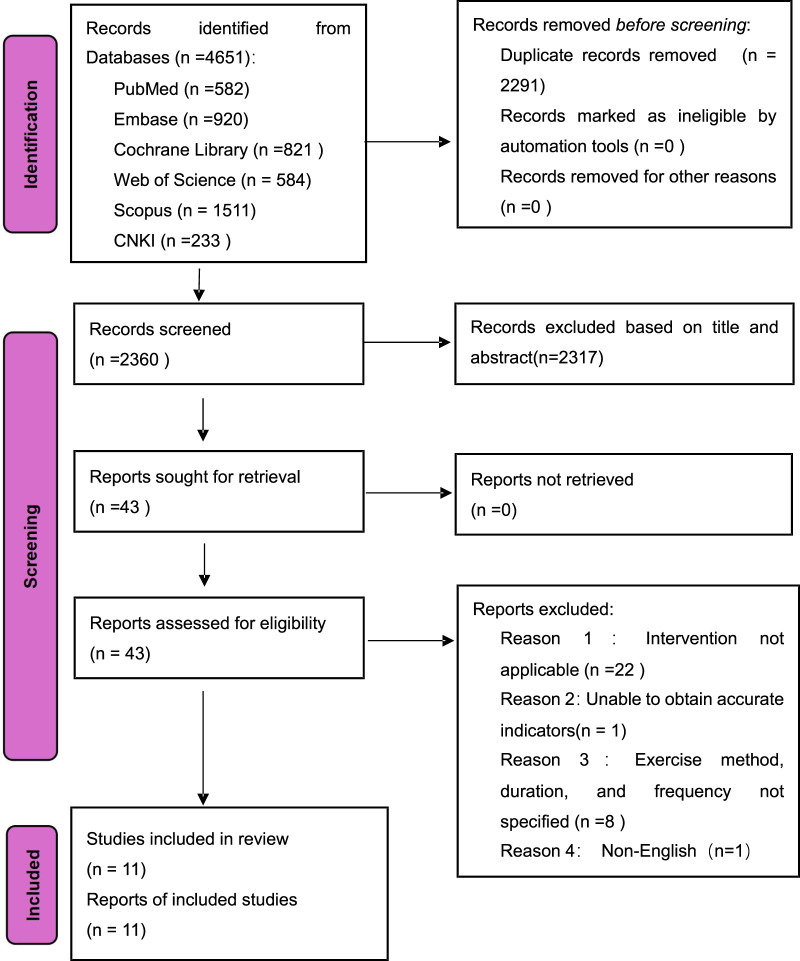
PRISMA flow chart for the present systematic reviews and meta-analysis.

In March 2025, we did a detailed search that resulted in 4,651 results from our database searches. After deleting duplicates, 2,360 unique records remained. By limiting the titles and abstracts, we were able to choose 43 full-text publications. Following a thorough review, we discovered 11 papers from 43 research that matched the established inclusion criteria. Figure 1 shows that 32 papers from these 11 studies were removed for a variety of reasons. Figure 1 shows a visual representation of our search approach using the PRISMA flow diagram. Our review comprises eleven randomized controlled trials. Table 1 present a summary of relevant participant characteristics.

**TABLE 1:** Characteristics of participants included in the study and exercise program and results of inclusion in the study.

Study details		Subjects				RT Intervention details						Whether dietary interventions	Main effects/outcomes		Compliance
Author, year	Country	Treatment Size(n)	Age (yr)	M/F	Inclusion criteria	Resistance type	Exercise session (mins)	Frequency (per wk)	Duration (weeks)	Intensity (%1RM)	Series (per-exercise)				
Hallsworth, 2011	United Kingdom	11	52	N/A	Adults Clinically diagnosed with non-progressive NAFLD Intrahepatic Lipid (IHL) content >5% NAFLD fibrosis score <1.445	8 exercises	45–60	3	8	50–70	3	No	1H-MRS-IHL	−1.80%	11/11
Lee, 2012	United States	16	14.6	16/0	Inclusion criteria Age: 12–18 years Gender: Male Developmental stage: Puberty (Tanner stage III-V) Body Mass index (BMI): ≥95th percentile (obese) Other: Non-smoker, non-diabetic, no participation in regular physical activity (except school physical education classes) in the past 3 months	10 exercises	60	3	12	60	3	YES	MRI-Intrahepatic lipid %	−1.20%	16/16
Bacchi, 2013	Italy	20	56	12/5	Age: 40–70 years Gender: Not limited Body Mass index (BMI): 24–36 kg/m^2^ Glycated hemoglobin (HbA1c): 6.5%–9.0% Disease status: Diagnosed with type 2 diabetes and non-alcoholic fatty liver disease (NAFLD) Other: No prior training, only using oral hypoglycemic drugs (excluding insulin)	9 exercises	N/A	3	16	70–80	3	YES	MRI - liver fat content	−11.30%	17/20
Jakovljevic, 2013	United Kingdom	9	49	7/2	Clinically diagnosed with NAFLD Intrahepatic Lipid (IHL) content >5% NAFLD fibrosis score <1.445	8 exercises	45–60	3	8	50–70	3	No	—	—	9/9
Zelber-Sagi, 2014	Israel	40	46.32	16/17	Age: 20–65 years Disease status: Diagnosed with fatty liver (NAFLD) via ultrasound Other: Diagnosed with fatty liver within the past 6 months and confirmed by baseline ultrasound examination	8 exercises	40	3	12	-	3	N/A	HRI	−0.25	36/40
Shamsoddini, 2015	Iran	10	45.9	10/0	Age: 32–54 years Gender: Male Disease status: Non-alcoholic fatty liver disease (NAFLD) confirmed by ultrasound, with intrahepatic triglyceride content greater than 5% Physical activity: No regular exercise habits	7 exercises	45	3	8	50–70	3	N/A	1H-MRI-IHL	−0.10%	10/10
Shelley E. Keating, 2017	Australia	15	45.4	2/13	Age: 29–59 years Gender: Not limited Physical activity level: Inactive (engaging in structured exercise less than 3 days per week, or moderate-intensity exercise less than 150 min per week) Body Mass index (BMI): BMI >25 kg/m^2^(overweight or obese) Disease status: No specific chronic diseases, but may have non-alcoholic fatty liver disease (NAFLD)	10 exercises	30–60	3	8	80–85	2–3	N/A	MRI-VAT	−175 cm^3^	12/15
Yao, 2018	China	34	55.8	16/16	Age: 18–75 years Disease status: Meets the diagnostic criteria of the 2010 edition of the ‘diagnosis and treatment guidelines for non-alcoholic fatty liver disease (NAFLD).' Muscle strength: Grade 5 muscle strength Exercise habits: No regular exercise Consciousness and communication skills: Conscious and capable of communication	Elastic Band	60	3	22	60–70	3	YES	Ultrasound	−0.6	32/34
Afsaneh Astinchap, 2021	United Kingdom	15	51	15/0	Age: 45–65 years Gender: Female Disease diagnosis: Diagnosed with type 2 diabetes and non-alcoholic fatty liver disease (NAFLD) Medical history: Diabetes history of at least 6 years BMI: 25–36 kg/m^2^ Blood glucose level: Fasting blood glucose between 120 and 150 mg/dL Glycated hemoglobin (HbA1c): 6.5%–9% Liver fat grade: Grade 2 or 3 fatty liver confirmed by ultrasound Medication use: Only metformin is allowed as a diabetes treatment drug	8 exercises	45–60	3	8	50–70	3	No	Ultrasound	−0.3	15/15
Phunchai charatcharoenwitthaya, 2021	Thailand	19	38.2	3/14	Subjects with non-alcoholic fatty liver disease (NAFLD) Age: Not explicitly mentioned, but subjects are adults Gender: Not explicitly mentioned, but subjects include both males and females Health status: Diagnosed with non-alcoholic fatty liver disease (NAFLD) Lifestyle: Sedentary lifestyle with no regular exercise habits Alcohol consumption: <20 g/d for females, <30 g/d for males Other: Agree to participate in the study and follow dietary and exercise interventions	10 exercises	60	5	12	60	3	YES	FibroScan	−13%	17/19
Irfan Varmazyar, 2024	Iran	10	31.6	N/A	Age: 18–45 years Body Mass index (BMI): 25–40 kg/m^2^ Disease status: Non-alcoholic fatty liver disease (NAFLD) confirmed by ultrasound Other: No other chronic liver diseases, such as hepatitis B, hepatitis C, or cirrhosis	8 exercises	40–45	3	12	60–80	3	N/A	Liver fat content%	−212.6%	10/10

### Basic information on the literature

The 11 included studies reported on the effects of resistance training on patients with MASLD. All 11 studies were English-language literature, and the study type was randomised controlled trials; they included 395 participants, with 203 in the resistance training group and 192 in the control group; Table 1 summarizes the characteristics of participants in the 11 included resistance exercise studies, covering literature from 2011 to 2024. The sample sizes of these studies ranged from 9 to 36 participants, with a wide age range, from a minimum of 14.6 years (Sojung et al., 2012) to a maximum of 56 years (Bacchi et al., 2013). Gender distribution varied significantly, with some studies predominantly male (Astinchap et al., 2021; Shamsoddini et al., 2015) and others predominantly female (Phunchai et al., 2021; Keating et al., 2017). Compliance rates were generally high, with most studies reporting good participant completion rates. Primary testing modalities included biochemical assays, imaging studies (e.g., MRI, ultrasound, Fibroscan), and magnetic resonance spectroscopy (1H-MRS), among others.

### Summary of bias risk assessment

**FIGURE 2:**
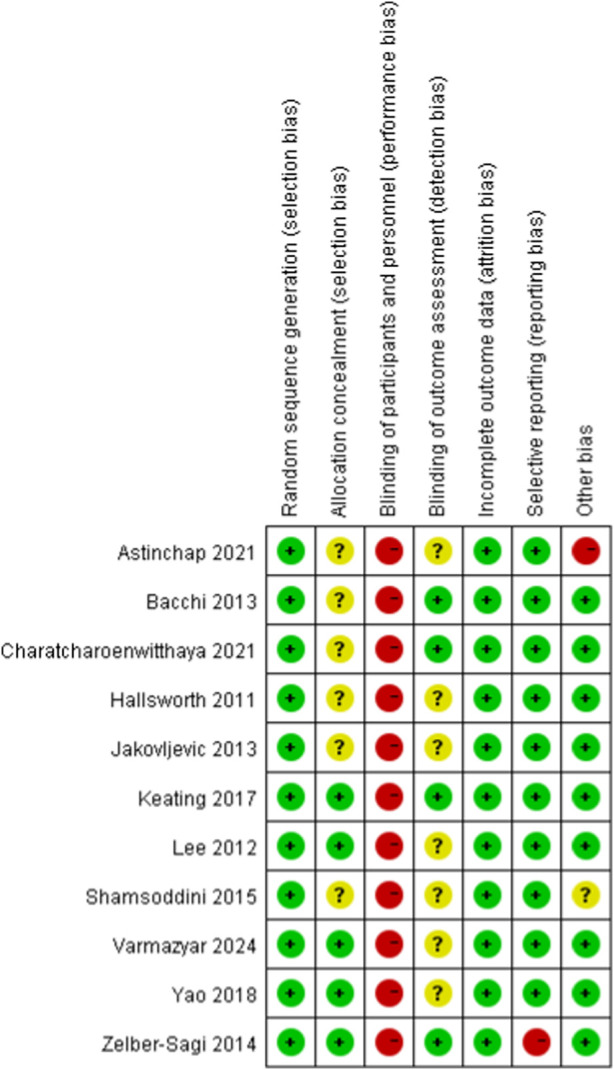
The results of the quality assessment of randomized controlled trials (RCTs).

**FIGURE 3:**
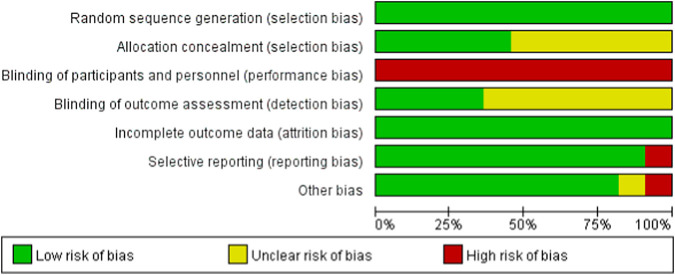
Risk of bias assessment of the included studies.

To evaluate the risk of bias in randomized controlled trials, we employed the Cochrane Randomized Trials Bias Risk Assessment Tool. Figures 2, 3 display the findings of the bias risk assessment. Because exercise interventions are unique, blinding of participants and implementers—a typical problem in this field—was not achievable in the 11 randomized controlled trials that made up this review. Furthermore, selection bias and other biases were found to have the highest risk of prejudice. Participants’ failure to follow the prescribed intervention procedure or the lack of proper analysis was the main cause of the high risk of selection bias. The high risk of other biases was attributed to concerns about data handling and discrepancies between the charts and their content.

### Exercise interventions

Our search identified 11 papers, all of which were randomized controlled trials that looked at the impact of resistance training interventions on liver lipid content and liver chemistry in patients with MASLD. Resistance training can be separated into two types based on the number of weeks of exercise: short-term (8 and 12 weeks) and long-term (16 and 22 weeks).

### Short-term resistance training

Shamsoddini et al. investigated the effects of aerobic and resistance exercise on liver fat content and enzyme levels in 30 patients with NAFLD (Shamsoddini et al., 2015). The resistance training group (n = 10) did seven exercises at an intensity of 50%–70% of maximum repetition (1RM) three times a week for 45 min each, while adhering to dietary restrictions. The final results revealed a decrease in ALT and AST levels, as well as a reduction in ultrasonography grades.

Keating et al. studied 15 overweight/obese people in a progressive resistance training research, with participants doing resistance exercises three times a week for 30–60 min each over an 8-week period (Keating et al., 2017). Volume and intensity were steadily raised from weeks 1-3, so that by week 4, three sets of 8–12 repetitions were accomplished at 80%–85% of 1-RM, with 60–120 s of rest between sets. Over the course of 8 weeks, the amount of exercise increased gradually as strength improved. Magnetic resonance spectroscopy (1H-MRS) and imaging were used to monitor and assess changes in liver fat.

The program consisted of ten exercises: seated leg press, chest press, lateral pull-down, calf raise, lunge, bicep curl, tricep dip, seated row, shoulder press, and abdominal sit-ups. The results revealed increased ALT and AST values. The 1H-MRS detection showed a 0.1% drop in IHL and a 175 cm^3^ reduction in VAT. The findings showed that standard progressive resistance training is unsuccessful at reducing liver fat in overweight/obese persons.

Astinchap et al. conducted a study on the effects of 8 weeks of endurance and resistance training on women with NAFLD and diabetes (Astinchap et al., 2021). Forty-five women with NAFLD and diabetes were randomly assigned to three groups: a control group (n = 15), an endurance training group (n = 15), and a resistance training group (n = 15). The resistance training protocol involved three sessions per week over 8 weeks, with each session consisting of three sets at 50%–70% of one-repetition maximum (1RM), 10–16 repetitions, and a duration of 35–50 min. In the first week, the training duration and intensity were 45 min and 50%, respectively. Training duration and intensity were increased weekly until they reached 60 min and 70%, respectively, by the eighth week. After 8 weeks of training, both endurance and resistance training were found to reduce AST and ALT levels. Moderate-intensity endurance training and resistance training both regulated the disruptive effects of type 2 diabetes and NAFLD on BKL and FGF-21 protein expression, with no significant differences between the two training methods.

Jakovljevic et al. carried out a randomized controlled experiment on 40 NAFLD patients aged 18–45 years (Jakovljevic et al., 2013). For a 12-week intervention, participants were randomly assigned to one of three groups: weight training (n = 14), moderate-intensity continuous aerobic exercise (n = 13), or usual care control (n = 13). Resistance training was done three times a week for 8 weeks. The program consisted of eight exercises: bicep curls, calf raises, tricep presses, chest presses, seated hamstring curls, shoulder presses, leg extensions, and lateral pulldowns. Each session lasted 45–60 min. The study found that the resistance training group had considerably lower ALT levels than the baseline. Structured resistance training successfully lowered liver enzyme levels and hepatic fat deposition in NAFLD patients while also improving metabolic function and lean body mass, potentially serving as an optimal intervention option independent of aerobic exercise.

Hallsworth et al. randomized sedentary persons with NAFLD to an 8-week resistance training program or continuing conventional treatment (Hallsworth et al., 2011). The resistance training regimen included three 45–60-min sessions each week with eight exercises: bicep curls, calf raises, tricep presses, chest presses, seated hamstring curls, shoulder presses, leg extensions, and lateral pulldowns. Each session lasted 45–60 min. In 1H-MRS, 8 weeks of resistance training lowered liver lipid levels by 13% and IHL levels by 1.8%, but had no effect on ALT levels. Resistance training had no effect on body weight, visceral fat tissue volume, or total body fat mass (p > 0.05). This is the first study to show that resistance exercise especially improves NAFLD, regardless of body weight.

Zelber-Sagi et al. conducted a randomized clinical trial in which 82 persons were identified with nonalcoholic fatty liver disease (NAFLD) using ultrasound (Zelber-Sagi et al., 2014). For 12 weeks, patients were randomly allocated to one of two groups: resistance training or control (home stretching activities). The resistance training regimen included three 40-min sessions each week, each with eight exercises: leg press, leg extension, leg curl, seated chest press, seated row, lat pulldown, bicep curl, and shoulder press. Each exercise was performed in three sets of 8–12 repetitions, with the training load gradually increasing based on individual capacity. The primary result was changes in liver fat content, which were measured using the hepatocystic index (HRI). The results showed that the resistance training group had significantly lower HRI scores, less overall fat, trunk fat, and abdominal fat, more lean body mass, and no link between weight changes and HRI improvement. Furthermore, ALT, AST, and GGT levels improved. This suggests that short-term resistance training can enhance liver fat content and metabolic indicators in NAFLD patients, independently of weight changes.

Varmazyar et al. performed a randomized double-blind controlled trial on 40 adult male patients diagnosed with non-alcoholic fatty liver disease (NAFLD) using ultrasound (Irfan et al., 2024). Over a 12-week intervention period, the resistance training program included eight exercises such as chest presses and high pulldowns that were performed three times weekly at an intensity of 60%–80% of one-repetition maximum (1RM), with 8–12 repetitions per set. Compared to the control group, the resistance training group had significantly lower body weight, BMI, trunk fat, and body fat percentage (all P < 0.05), better muscle strength (1RM), and lowered ALT and AST values. The study found that 12 weeks of resistance training successfully improved body composition, metabolic abnormalities, and liver inflammation in NAFLD patients, whereas vitamin E supplementation had just a little effect on liver enzymes.

Lee et al. conducted a randomized controlled experiment with 45 obese male adolescents aged 12–18 years to determine the effects of resistance training on abdominal fat, hepatic lipids, and insulin sensitivity (Sojung et al., 2012). Participants were randomly assigned to either the resistance training, aerobic activity, or control groups. The resistance training regimen featured eight full-body exercises (such as leg press, leg extension, and leg curl). During the first 4 weeks, individuals used correct weightlifting technique to complete one to two sets of 8–12 repetitions at 60% of their baseline one-repetition maximum (RM). From weeks 4–12, participants completed two sets of 8–12 repetitions until failure. MRI scans of visceral fat alterations demonstrated a reduction in hepatic fat following resistance training. The study revealed that resistance training substantially lowers visceral fat and hepatic lipid deposition in obese teenagers without calorie restriction while also dramatically improving insulin sensitivity through muscle building. Although all exercise styles are effective for abdominal obesity, resistance training has more metabolic benefits. Furthermore, participants in the resistance training group showed higher compliance (99% attendance rate) and more favorable subjective experiences, indicating that it may be better suited for long-term health interventions in teenagers.

Charatcharoenwitthaya et al. conducted a randomized controlled experiment on 35 adult NAFLD patients to determine the efficacy of resistance training combined with dietary changes (Phunchai et al., 2021). Participants were randomly assigned to one of two groups: 12-week moderate-intensity weight training (n = 17) or moderate-intensity aerobic exercise (n = 18), with both groups receiving monthly dietary adjustments advised by a dietitian. The resistance training regimen consisted of five 60-minute sessions each week, each with eight resistance exercises (for example, leg press, bench press, lat pulldown, etc.). Additionally, single-set push-ups and sit-ups were done. During the first 4 weeks, participants used correct lifting technique to complete 1 or 2 sets of 8–12 repetitions at 60% of their one-repetition maximum (1RM). From weeks 5–12, individuals performed two sets of eight to twelve repetitions at 60% of their 1RM until failure. The results demonstrated that resistance training dramatically enhanced muscle insulin sensitivity index and lowered visceral fat scores, with benefits similar to aerobic exercise. Furthermore, moderate-intensity resistance training combined with nutritional intervention substantially reduced liver fat content and increased insulin sensitivity in NAFLD patients, producing results comparable to aerobic exercise. The study underscores how strength training’s increase in muscle metabolism may indirectly regulate hepatic lipid metabolism, providing an alternate option for those who cannot handle aerobic exercise.

### Long-term resistance training

Bacchi et al. conducted a randomized controlled trial involving 31 patients with type 2 diabetes and MASLD (Bacchi et al., 2013). The primary objective of the study was to compare the effects of aerobic exercise versus resistance training on insulin sensitivity, body composition, and liver fat content, as well as visceral, subcutaneous, and deep subcutaneous fat tissue. Liver fat content was measured using MRS in the resistance training group (n = 17). Throughout 16 weeks, the resistance training group engaged in three sets of 10 repetitions each at 70%–80% of 1RM while following a controlled diet. They performed nine different exercises on a weight machine (chest press, shoulder press, vertical pull, leg press, leg extension, leg curl, and abdominal crunch) and with free weights (biceps, abdominal) three times a week. According to the data, the resistance training group’s ALT and GGT levels dropped but their AST levels stayed mostly constant. Additionally, the absolute value of liver fat content significantly fell. Resistance training and aerobic exercise are equally efficient in lowering liver fat content in people with type 2 diabetes and non-alcoholic fatty liver disease (NAFLD), according to the first randomized controlled experiment.

Yao et al. investigated the effects of aerobic and resistance training on ALT and cholesterol levels in 103 patients with NAFLD (Yao et al., 2018). 103 NAFLD patients were randomly assigned to aerobic exercise (n = 34), resistance exercise (n = 34), or a non-exercise control group (n = 35) for a 22-week intervention. All patients were given dietary information. In the resistance exercise group (n = 31), elastic bands were used three times a week for 60 min each, with three sets at 60%–70% of 1RM intensity and ten repetitions per set. After 22 weeks of exercise, the resistance training group’s ALT levels reduced by 6.15 U/L. Both the aerobic and resistance training groups demonstrated notable increases in HDL when compared to the control group. According to this study, Chinese NAFLD patients’ HDL can be successfully raised by both resistance and aerobic exercise.

### Meta-analysis

#### Clinical compliance

**FIGURE 4:**
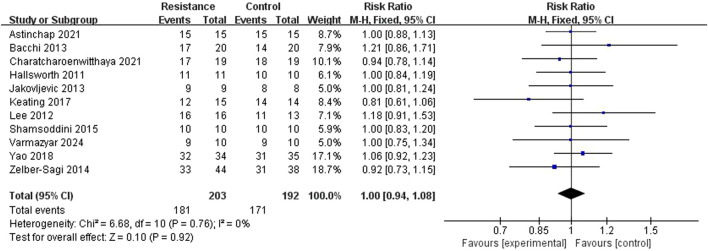
A meta-analysis forest plot comparing clinical compliance between the two patient groups.

**FIGURE 5:**
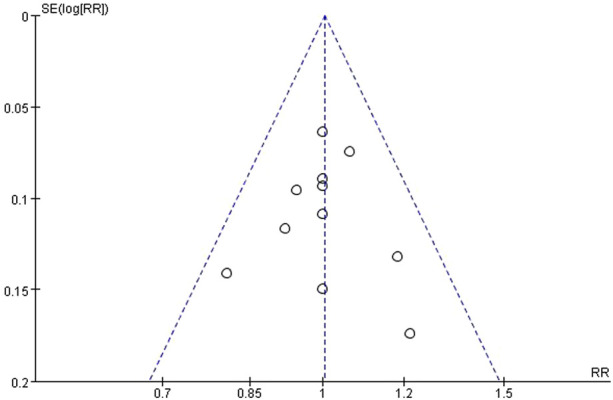
Meta-analysis funnel diagram evaluating clinical compliance between two patient groups.

The meta-analysis comprised 11 RCT studies reporting clinical compliance, totaling 395 participants, with comparable sample sizes in the resistance exercise and control groups. The studies showed low heterogeneity (I^2^ = 0%, P = 0.76), hence a fixed-effect model was used. The analysis results showed no statistically significant difference in compliance between the resistance exercise group and the control group (RR = 0.99, 95% CI = 0.89 to 1.10, Z = 0.10, P = 0.92), indicating no significant difference in the incidence of compliance events between the two groups (Figures 4, 5). A sensitivity analysis, removing one study at a time, confirmed the stability of this result.

#### Alanine aminotransferase (ALT)

**FIGURE 6:**
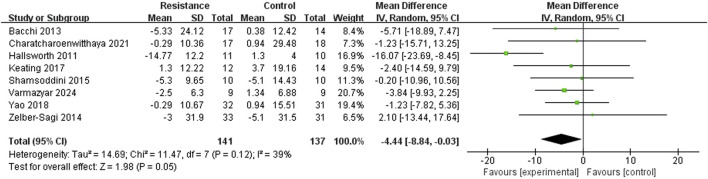
Forest plot of ALT improvement comparison between groups.

Eight studies (Hallsworth et al., 2011; Phunchai et al., 2021; Keating et al., 2017; Shamsoddini et al., 2015; Zelber-Sagi et al., 2014; Irfan et al., 2024; Sojung et al., 2012) reported ALT levels to determine the influence of resistance training on blood alanine aminotransferase. The studies showed moderate heterogeneity (I^2^ = 39%, P = 0.12). A random-effects model was used as a conservative measure. The meta-analysis revealed a statistically significant difference in ALT changes between the resistance exercise and control groups (MD = −4.44 U/L, 95% CI = −8.84 to −0.03, Z = 1.98, P = 0.05), indicating that the resistance exercise group outperformed the control group in lowering ALT levels (Figure 6). Sensitivity analysis showed that the result remained statistically significant (P < 0.05) upon sequential removal of any single study, reinforcing the finding.

#### Aspartate aminotransferase (AST)

**FIGURE 7:**
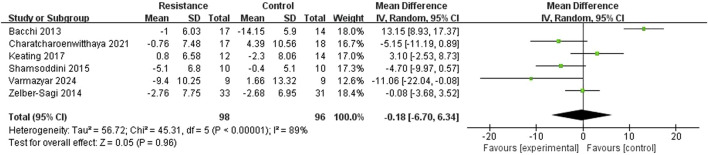
Forest plot of AST level comparison between groups.

Six studies (Bacchi et al., 2013; Phunchai et al., 2021; Keating et al., 2017; Shamsoddini et al., 2015; Zelber-Sagi et al., 2014; Irfan et al., 2024)reported AST levels. There was high heterogeneity among the studies (I^2^ = 89%, P < 0.00001), so a random-effects model was selected. The results showed that there was no statistically significant difference in AST changes between the resistance training group and the control group (MD = −0.18 U/L, 95% CI = −6.70 to 6.34, Z = 0.05, p = 0.96), indicating that the resistance training group did not show a significant difference in reducing AST levels compared to the control group (Figure 7).

#### Other potential parameters for meta-analysis

We attempted to perform meta-analyses on other clinically relevant outcomes, including body weight, BMI, gamma-glutamyl transferase (GGT), and quantitative liver fat content measured by imaging. However, a meaningful statistical synthesis was precluded for these outcomes due to insufficient reported data (e.g., missing standard deviations) or high heterogeneity in measurement methods across studies (e.g., liver fat assessed by ultrasound, MRI, or FibroScan without interchangeable units). Future studies with standardized reporting of these outcomes are needed to evaluate the effects of resistance training more comprehensively.

## Discussion

The minimum effective dose for patients with aberrant ALT levels or those who cannot handle aerobic activity should be at least three sessions per week, lasting roughly 60 min each, for a minimum of 12 weeks, according to an analysis of 11 research. Workouts that work several muscle groups across the body are the best way to control the intensity, which should be between 60% and 80% of 1RM (8–10 workouts are advised). Leg press, high pulldown, biceps curl, leg extension, shoulder press, chest press, triceps pushdown, triceps extension, leg curl, and calf raise are the ten exercises that are most frequently performed. The patient’s condition should be tracked in real time while exercising, and the progressive progression and individualization principles should be adhered to.

Resistance training is a good substitute for aerobic exercise for those who have trouble with it. The review’s meta-analysis findings, however, indicate that although the resistance training group’s ALT levels were lower than the control group’s, there was a large degree of variability and no discernible change in their AST levels. The limited number of included studies and small sample sizes may be the cause of this, as it is challenging to ascertain the precise impact of resistance exercise on AST. The success of the intervention may also be impacted by individual patient variations, including age, gender, and preexisting health conditions.

Regarding diet, four trials demonstrated that dietary management improved biochemical and imaging markers associated with MASLD. The Mediterranean diet has a significant effect on MASLD, according to other research, although it must be modified to fit a Chinese dietary pattern.

Imaging markers, liver biochemical markers, and liver biopsies are presently the main outcome measures in use. However, fatty degeneration cannot be accurately measured using liver biochemical markers alone. The histological significance of AST in MASLD is still unknown, despite the fact that ALT alterations have been demonstrated to correlate with histological improvement in MASLD patients (Di Martino et al., 2016; Newton et al., 2021), Although liver biopsy is the clinical reference standard for hepatic steatosis diagnosis, its application in long-term clinical trials is limited due to its invasiveness.

Liver biopsies were not used in any of the 11 investigations in this evaluation. MRI and MRS are known for their accuracy, safety, and reproducibility among non-invasive imaging options. Both MRI and MRS have established diagnostic thresholds and shown accuracy in evaluating hepatic steatosis (Pasanta et al., 2021; Di Martino et al., 2016; Middleton et al., 2018; Schwimmer et al., 2015; Go et al., 2020). However, not all studies contain pertinent imaging tests because of their high cost.

## Advantages and limitations

The benefits of our systematic review derive from our extensive and rigorous strategy, which included systematic searches of six databases and a study of the reference lists of primary research and relevant reviews to guarantee a thorough analysis of the available literature. Furthermore, two review writers independently examined each paper, which improved the quality of our analysis. We focused on the effects of exercise therapies on measurable clinical markers associated with MASLD.

In contrast to other literature reviews, this study used the most recent nomenclature, “metabolic dysfunction-associated steatohepatitis (MASLD),” which reflects a stronger understanding of the condition and is in line with the current international naming consensus (e.g., the 2023 name update). It improves clinical applicability by covering a larger population with metabolic disorders. A more strong evidence foundation was provided by the review, which comprised 11 randomized controlled trials (RCTs) with a bigger sample size (203 cases) than the 2016 study, which only included 4 RCTs (135 cases), extended the time period to 2025, and examined more databases (e.g., Scopus, Web of Science). To lower the possibility of bias and increase the validity of the findings, non-randomized studies were specifically disregarded.

This review has several limitations. First, the included studies did not report on liver fibrosis outcomes, such as changes in elastography or histologic fibrosis staging. Therefore, the effect of resistance training on fibrosis regression or progression in MASLD remains unknown. This is a critical gap, as fibrosis stage is a key predictor of liver-related outcomes in MASLD. Future randomized trials should incorporate non-invasive fibrosis markers (e.g., VCTE, FIB-4, ELF) or liver biopsy to address this question. Language and publishing bias may be introduced by excluding unpublished and non-English studies. Furthermore, the inclusion of only 11 research yielded a limited number of papers, thereby limiting the diversity and generalizability of the data.

## Current literature and recommendation

Given that liver disease is still difficult to manage despite a variety of treatment choices, it is imperative to comprehend how resistance exercise affects MASLD patients. Exercise is a powerful tool for preventing and treating liver illness as well as for preserving and reestablishing body balance (Zhang et al., 2024). As of right now, lifestyle changes remain the mainstay of managing MASLD because there are no specific targeted medication therapy for the condition. According to the American Clinical Assessment and Management Practice Guidelines, MASLD can be prevented or improved by increasing activity levels by more than 60 min per week, or by participating in moderate-intensity exercise at least five times per week for a total of 150 min (Rinella et al., 2023). Nevertheless, the evidence basis for resistance training is small, and there are no precise recommendations for the precise exercise techniques, frequency, duration, or workout cycles. According to some research, exercise can reduce liver fat, and the best kind of exercise is a combination of weight training and aerobic activity (Xue et al., 2024). Resistance training, however, becomes the recommended form of exercise for individuals who find aerobic activity inconvenient. Nine studies showed that resistance training can reduce liver fat, and the results of eleven studies on the effects of resistance training on MASLD were compiled in this review. Resistance training shows promise as a workable therapy option for MASLD as study continues. However, more information about the precise resistance training techniques is required.

## Conclusion

For patients with unusually elevated ALT levels who are unable to perform aerobic exercise (such as those with joint problems or poor cardiopulmonary function), resistance training is a useful addition to lifestyle therapies for MASLD. To prevent overloading injuries, real-time movement supervision is emphasized during training. The impact of resistance training on biochemical and imaging markers in patients with MASLD is summed up in this review. But there are still a number of unanswered questions. 1) Long-term research on resistance training in MASLD is lacking; 2) meta-analyses indicate that resistance training may raise ALT levels, but its effects on AST are still unknown; 3) little is known about how dietary control affects MASLD. To better understand the benefits of resistance exercise on its own, future research should create large sample sizes, high-quality controlled trials that account for dietary factors.

## Data Availability

The original contributions presented in the study are included in the article/supplementary material, further inquiries can be directed to the corresponding authors.
