# Supplementary material for: Resistance training for metabolic dysfunction-associated steatotic liver disease:a systematic review and meta-analysis
Source: Front Physiol. 2026 Feb 2;16:1679094. doi: 10.3389/fphys.2025.1679094 (PMC12907158; doi:10.3389/fphys.2025.1679094)
Supplement: Supplementary file 2 [file DataSheet4.pdf]

NIHR | National Institute for Health and Care Research

PROSPERO
International prospective register of systematic reviews

Effects of Resistance exercise on Patients with Metabolic Dysfunction-Associated Steatotic Liver Disease: A Systematic Review

yun chen, xiaoya qiao, xinyi zou

Citation
yun chen, xiaoya qiao, xinyi zou. Effects of Resistance exercise on Patients with Metabolic Dysfunction-Associated Steatotic Liver Disease: A Systematic Review . PROSPERO 2025 CRD420251050504. Available from https://www.crd.york.ac.uk/PROSPERO/view/CRD420251050504.

REVIEW TITLE AND BASIC DETAILS

Review title

Effects of Resistance exercise on Patients with Metabolic Dysfunction-Associated Steatotic Liver Disease: A Systematic Review

Condition or domain being studied

Resistance training ; Non-alcoholic fatty liver disease

Rationale for the review

Metabolic dysfunction-associated steatotic liver disease (MASLD) has emerged as the most prevalent chronic liver disease globally, affecting over 20% of the adult population worldwide, with a notably high prevalence of 29.2% in the Chinese population. This condition is not only strongly associated with an elevated risk of hepatic complications, including cirrhosis and hepatocellular carcinoma, but also intricately linked to systemic metabolic disorders such as metabolic syndrome, cardiovascular diseases, and chronic kidney disease. Current management guidelines emphasize lifestyle interventions—including weight reduction, dietary modifications, and physical activity—as foundational therapeutic strategies, while pharmacological therapies remain under clinical development. Although moderate-intensity aerobic exercise is endorsed by clinical guidelines as an effective modality for ameliorating hepatic steatosis, the therapeutic efficacy of resistance exercise in MASLD remains inadequately characterized. Resistance exercise may serve as a viable alternative for patients with compromised cardiopulmonary function or limited capacity for aerobic activities. However, its impact on hepatic health lacks robust systematic evidence. This systematic review aims to address this critical knowledge gap and provide an evidence-based foundation for formulating resistance exercise protocols tailored to MASLD patients.

Review objectives

The primary objective of this review is to investigate the impact of resistance exercise on liver disease outcomes in patients with metabolic dysfunction-associated steatotic liver disease (MASLD). Considering the evolving terminology in this field and acknowledging that most included studies were published prior to recent nomenclature updates, we have retained the original terms "non-alcoholic fatty liver disease (NAFLD)" and "metabolic-associated fatty liver disease (MAFLD)" throughout this review to maintain consistency with the source literature. We conducted a comprehensive systematic review to elucidate the effects of resistance exercise interventions on hepatic health parameters in MASLD. This synthesis aims to critically evaluate existing evidence regarding the role of resistance training in managing metabolic dysfunction-associated steatotic liver disease, thereby establishing a theoretical foundation for developing optimized resistance exercise protocols tailored for MASLD populations.

Keywords

Resistance exercise; Metabolic dysfunction-associated steatotic liver disease MASLD; Fatty liver

Country

China

ELIGIBILITY CRITERIA

Population

Included

Inclusion criteria: ① Patients diagnosed with MASLD or NAFLD; ②Outcome measures: Quantitative imaging assessment of hepatic steatosis, hepatic biochemistry, or hepatic histology must be included;

Excluded

Exclusion criteria were any known secondary liver disease including the presence of hepatitis B surface antigen or anti-hepatitis C virus antibodies, excessive alcohol consumption defined as ≥ 30 g/d in men or 20 g/d in women, administration of medical treatment that may elevate alanine aminotransferase (ALT) or lead to hepatic steatosis, known diabetes, major chronic diseases including: renal, cardiovascular, lung, uncontrolled hypertension, inflammatory bowel disease, active cancer, autoimmune disorders and orthopedic contraindications for RT.

Intervention(s) or exposure(s)

Included

Resistive Exercise; Resistance training

Comparator(s) or control(s)

This review does not have any comparators

Study design

Only randomized study types will be included.

Context

The intervention must be clearly defined as resistance exercise, with no restrictions on the location of the intervention.

TIMELINE OF THE REVIEW

Date of first submission to PROSPERO
12 May 2025

Review timeline
Start date: 1 May 2025. End date: 1 December 2027.

Date of registration in PROSPERO
12 May 2025

AVAILABILITY OF FULL PROTOCOL

Availability of full protocol
A full protocol has been written and uploaded to PROSPERO. The protocol will be made available after the review is completed.

SEARCHING AND SCREENING

Search for unpublished studies
Only published studies will be sought.

Main bibliographic databases that will be searched
The main databases to be searched are *CENTRAL - Cochrane Central Register of Controlled Trials*, *Embase.com*, *PubMed* and *Scopus*.

Other important or specialist databases that will be searched
Web of Science and CNKI

Search language restrictions
The review will only include studies published in English.

Search date restrictions
There are no search date restrictions.

Other methods of identifying studies
No other methods will be used.

Link to search strategy
A full search strategy is available in the full protocol as described in the *Availability of full protocol* section

Selection process
Studies will be screened independently by at least two people (or person/machine combination) with a process to resolve differences.

Other relevant information about searching and screening
None

DATA COLLECTION PROCESS

Data extraction from published articles and reports

Data will be extracted independently by at least two people (or person/machine combination) with a process to resolve differences.

Authors will not be contacted for further information.

Study risk of bias or quality assessment

Risk of bias will be assessed using: *Cochrane RoB-2*

Data will be assessed independently by at least two people (or person/machine combination) with a process to resolve differences.

Additional information will **not** be sought from study investigators if required information is unclear or unavailable in the study publications/reports.

Reporting bias assessment

Risk of bias due to missing results will be assessed

Certainty assessment

The reviewers further used Version 1 of the Cochrane risk-of-bias tool for randomized trials (RoB 2) to assess the risk of bias for each outcome of the included RCTs through the following aspects： randomization process, deviations from intended interventions, missing outcome data, measurement of the outcome, and selection of the reported result ,Each indicator contains three levels: low risk, unclear and high risk.

OUTCOMES TO BE ANALYSED

Main outcomes

1.Quantitative imaging assessment of hepatic steatosis

2.Liver chemistries (ALT and GGT)

3.Changes in liver histology

Additional outcomes

There are no additional outcomes.

PLANNED DATA SYNTHESIS

Strategy for data synthesis

No formal data synthesis is planned - data will be described but not combined.

CURRENT REVIEW STAGE

Stage of the review at this submission

Review stage	Started	Completed
Pilot work	✓	
Formal searching/study identification	✓	
Screening search results against inclusion criteria	✓	

Review stage	Started	Completed
Data extraction or receipt of IPD		
Risk of bias/quality assessment		
Data synthesis		

Review status
The review is currently planned or ongoing.

Publication of review results
Results of the review will be published.

REVIEW AFFILIATION, FUNDING AND PEER REVIEW

Review team members
Mrs yun chen (review guarantor and contact) Nanjing Institute of Physical Education. China.

No conflict of interest declared.

Mrs xiaoya qiao. Nanjing Institute of Physical Education. China.

No conflict of interest declared.

Mrs xinyi zou. Nanjing Institute of Physical Education. China.

No conflict of interest declared.

Named contact
Mrs yun chen (1540405342@qq.com). Nanjing Institute of Physical Education. China.

Review affiliation
Nanjing Institute of Physical Education

Funding source
Review has no funding and no agreed support from an academic institution and is done in authors' own time.

Peer review
There has been no peer review of this planned review.

ADDITIONAL INFORMATION

Review conflict of interest
Declared individual interests are recorded under team member details.. No additional interests are recorded for this review.

Medical Subject Headings
Non-alcoholic Fatty Liver Disease

SIMILAR REVIEWS

Check for similar records already in PROSPERO

PROSPERO identified a number of existing PROSPERO records that were similar to this one (last check made on 12 May 2025). These are shown below along with the reasons given by that the review team for the reviews being different and/or proceeding.

- Metabolic dysfunction-associated steatotic liver disease (MASLD) and risk of Chronic Kidney Disease [published 17 April 2024] [CRD42024531735]. The review was judged **not to be similar**
- Lipoprotein(a) circulating levels and steatotic liver disease related to metabolic dysfunction: a systematic review and meta-analysis [published 30 October 2024] [CRD42024607750]. The review was judged **not to be similar**
- Circulating Angiopoietin-like protein 8 (ANGPTL8) and steatotic liver disease related to metabolic dysfunction: a systematic review and meta-analysis [published 10 September 2024] [CRD42024584720]. The review was judged **not to be similar**
- Navigating the Effects of Resistance Training on Patients with Metabolic Dysfunction-Associated Steatotic Liver Disease: A Systematic Review and Meta-Analysis [published 3 May 2025] [CRD420251045280]. The review was acknowledged as **similar** but the authors opted to continue because *the review will be more up to date*, *the review looks at additional or different outcomes*

PROSPERO version history

- Version 1.0, published 12 May 2025

Disclaimer

The content of this record displays the information provided by the review team. PROSPERO does not peer review registration records or endorse their content.

PROSPERO accepts and posts the information provided in good faith; responsibility for record content rests with the review team. The guarantor for this record has affirmed that the information provided is truthful and that they understand that deliberate provision of inaccurate information may be construed as scientific misconduct.

PROSPERO does not accept any liability for the content provided in this record or for its use. Readers use the information provided in this record at their own risk.

Any enquiries about the record should be referred to the named review contact
