# Supplementary figures and images for: Resistance training for metabolic dysfunction-associated steatotic liver disease:a systematic review and meta-analysis
Source: Front Physiol. 2026 Feb 2;16:1679094. doi: 10.3389/fphys.2025.1679094 (PMC12907158; doi:10.3389/fphys.2025.1679094)

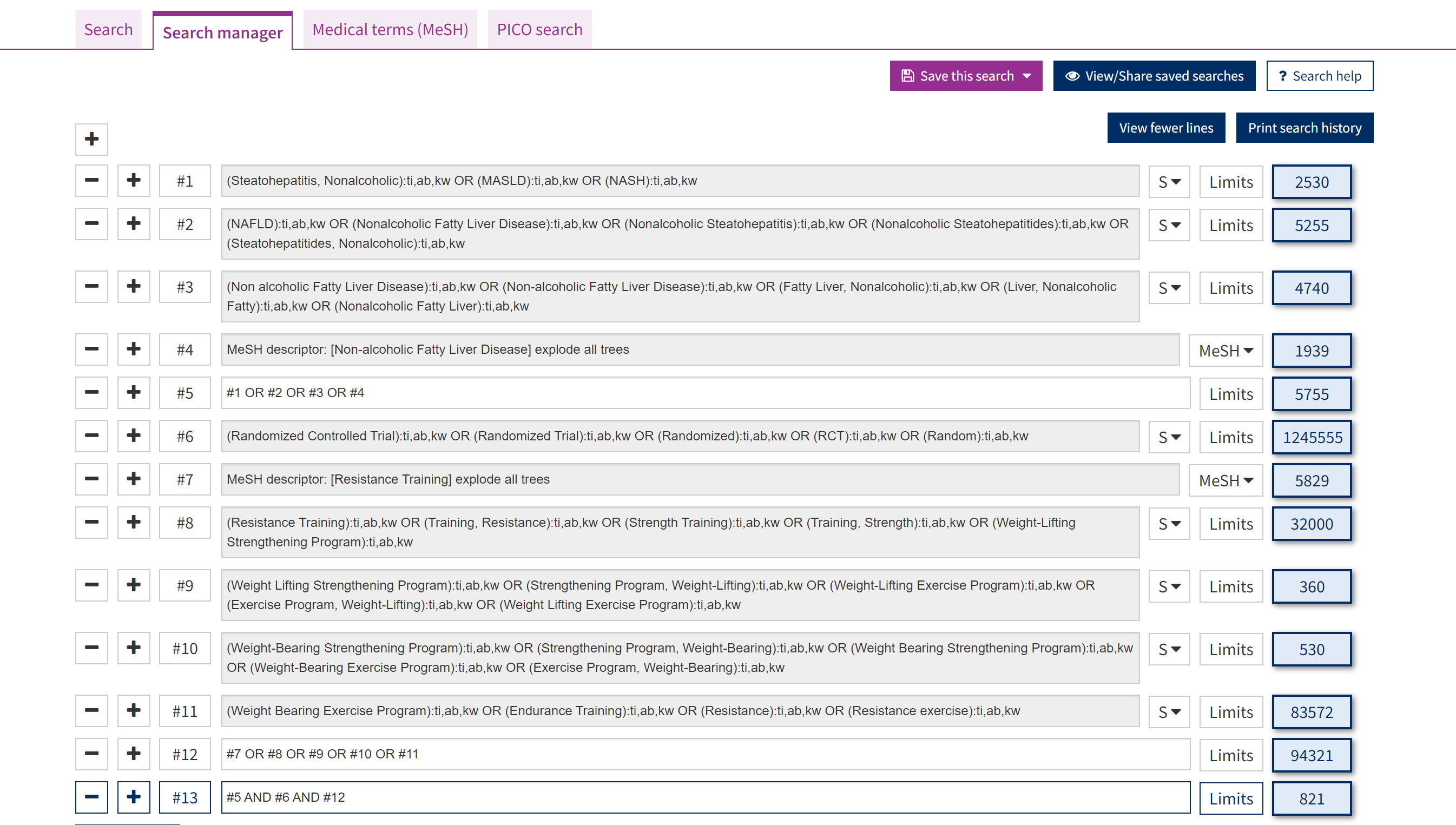

Supplement: Supplementary file 5 [file Image1.png]
